# Feasibility and Safety of Single-Incision Laparoscopic Surgery in Gastric Benign and Malignant Diseases

**DOI:** 10.1155/2014/782035

**Published:** 2014-05-28

**Authors:** Kong Jing, Wu Shuo-dong, Fan Ying, Wang Yu

**Affiliations:** Department of Minimally Invasive Surgery, Shengjing Hospital, China Medical University, Sanhao Street No. 36, Shenyang, Liaoning 110004, China

## Abstract

We systematically searched Medline and Cochrane Library and the related references occurred in the citations until February 2013 and included all the cases who suffered from gastric benign and malignant diseases where single incision laparoscopic surgery was performed. A total of 69 cases with gastric diseases undergoing SILS surgery were reviewed to evaluate the feasibility, safety, and potential benefits of SILS for the gastric diseases. Demographic data, morphologic characterisation of the lesion, and perioperative parameters were analyzed retrospectively. The initial experience with SILS for benign gastric diseases and early gastric cancer showed that it is feasible and safe when performed by experienced laparoscopic surgeons. Despite the limited number and technical difficulties it can be proposed in selected patients.

## 1. Introduction

Laparoscopic gastrectomy is being accepted as a safe and feasible surgical procedure for a wide range of gastric benign diseases owing to its less invasive and comparable oncologic outcomes. Since the first laparoscopy-assisted distal gastrectomy reported by Kitano et al. in 1994 [1], laparoscopic radical gastrectomy (LADG) for early gastric cancer has also become a widely accepted procedure. Some randomized control studies (RCTs) have clearly demonstrated short-term benefits including early postoperative recovery, decreased postoperative pain, shorter hospitalization, and similar number of harvested lymph nodes [2–5]. Laparoscopic surgery typically uses three or four ports for gastric procedure, with more potential morbidity from port-site bleeding, hernia, and infection. In recent years, laparoscopic surgery has become even less invasive with the introduction of transumbilical single-incision surgery due to its cosmetic advantage. It has been used for various cholecystectomies, appendectomies, nephrectomies, and other related surgical operations, and recently this technique has been applied in gastric surgery. Because of the limited number of studies reported about SILS in gastric diseases, its clinical significance remains to be elucidated. The purpose of this review is to assess the feasibility and safety of SILS for gastric diseases.

## 2. Materials and Methods

### 2.1. Literature Search Strategies

A systematic search of the scientific literature was carried out using the online databases (PubMed and the Cochrane library) and the related references that occurred in the citations to identify any articles missed by the initial search were manually searched up to February 1, 2013. The search terms were “single incision,” “single port,” “single access,” “single site,” “laparoscop*,” “laparoendoscop*,” “peritoneoscop*,” “stomoch*,” “gastric,” “gastrectomy,” and “gastro*.” There were no language restrictions during the search and the search strategy in this study was listed as below:

#14 Add Search (((#13) OR #12) OR #11) OR #10

#13 Add Search gastro*

#12 Add Search gastrectomy

#11 Add Search gastric

#10 Add Search stomoch*

#9 Add Search ((#8) OR #7) OR #6

#8 Add Search peritoneoscop*

#7 Add Search laparoendoscop*

#6 Add Search laparoscop*

#5 Add Search (((#4) OR #3) OR #2) OR #1

#4 Add Search single site

#3 Add Search single access

#2 Add Search single port

#1 Add Search single incision.

### 2.2. Inclusion Criteria

Articles were selected if the abstract contained data of patients who underwent SILS for gastric diseases in all the forms, such as RCTs, other controlled or retrospective studies, and case reports. The references of these articles were also reviewed in order to find additional relevant studies. Only the latest and most complete study was included when the duplicate cases in different publications were reviewed. Reports with animal model in SILS, review articles, and studies about different SILS for the treatment of obesity were excluded from this study. The full text provided that the data for this review were verified by each of the two investigators (JK and YF) who independently decided on inclusion/exclusion and independently abstracted the study data. Disagreements and discrepancies were resolved by consensus.

## 3. Results

Up to February 1, 2013, a total of 69 cases with gastric diseases underwent SILS surgery. Among these, 7 studies with 31 patients undergoing SILS partial resection of the stomach or gastric wedge resection were reviewed. SILS subtotal gastrectomy was performed in 15 patients introduced by 5 studies, respectively. And in 4 of these 5 studies, the lymphadenectomy was also performed for 14 patients with early gastric cancer. A case about SILS perforated gastric ulcer repair was reported. Furthermore, the method of intracorporeal circular-stapled Billroth I anastomosis for 20 cases that underwent SILS distal gastrectomy was introduced in 1 study. Another method for intracorporeal anastomosis for gastrojejunostomy was performed in 2 cases with gastric outlet obstruction.

### 3.1. SILS in Partial Gastrectomy for Submucosal Tumors

A total of 7 studies with 31 patients undergoing SILS partial resection of the stomach or gastric wedge resection were reviewed [6–12] (Tables 1 and 2). In Na et al.'s report [6], 7 patients diagnosed as gastric submucosal tumor underwent the intragastric wedge resection. Among these 31 cases, 27 GISTs [6–12], 1 nonspecific inflammatory [12], 2 leiomyoma [6], and 1 benign carcinoma tumor [11] were confirmed by the final pathology. 11 tumors were located on the fundus of stomach [6, 7, 12], 8 tumors located on the body of the stomach [6, 7, 11, 12], 4 tumors located on the anterior wall [7, 8, 11], 3 tumor located on the posterior wall [7, 9], and 5 tumors located on the gastric cardia [6, 10]. Pathology confirmed the diameter of tumors from 0.6 cm to 5.4 cm (<2 cm in 5 cases and ≥2 cm in 26 cases). Though flexible laparoscope and curved or double-bended graspers were used frequently and made the SILS operation more easily, conventional laparoscopic instruments can achieve the similar clinical outcomes [7]. Also an extra mini-port for 2 mm mini-loop was often used to retract the liver or gastric wall surrounding the tumor to facilitate the dissection and resection [8–11]. Ranges of all the operative times were from 64 to 147 minutes and estimated blood losses were minimal. There were no cases converted to conventional (multiport) laparoscopic or open operations in all the above patients. Except wounding bleeding in one case [6], no intraoperative or other postoperative complications were experienced in all the patients including anastomotic leakage, bleeding, or anastomotic stenosis.

### 3.2. SILS in Distal Gastrectomy for Peptic Ulcer and Early Gastric Cancer

SILS subtotal gastrectomy was performed in 15 patients introduced by 5 studies, respectively [13–17]. And in 4 studies, the lymphadenectomy was also performed for 14 patients with early gastric cancer [13–16] (Tables 3 and 4).

A patient with an intractable gastric ulcer was treated by SILS gastrectomy with intracorporeal Roux-en-Y reconstruction [17]. In this patient, a 3 cm hernial orifice was seen between the duodenum and the retroperitoneal area. A large opening was made in the hernial orifice by mobilizing the right-side colon. The appendix was also excised is this patient. The operative time was 412 min, with blood loss of 90 mL. An additional 2 mm mini-loop retractor was inserted through an extra incision in the right subcostal space to create the operative field. The patient started a normal diet on day 3 and was discharged on postoperative day 14 with no complications.

14 patients underwent single-incision laparoscopic distal gastrectomy and lymph node D1*α* or D1*β* or D2 dissection in 4 reports [13–16]. Computed tomography showed that there was no regional LN or distant metastases before the operation. The final pathology report showed that the tumor had invaded the submucosa or proper muscle and with no seromuscular layer infiltration. A total of 236 lymph nodes were retrieved. Except one patient with 5 metastases among dissected 21 LNs [14] and one patient with 1 metastasis [16], lymph nodes of other patients were identified with no tumor metastasis. Proximal and distal resection margins were free of tumor. Gastroduodenostomy was performed in 8 patients [13, 14] and 5 cases with gastrojunostomy [15, 16] and 1 case with Roux-en-Y reconstruction [13]. The range of the operative time was from 140 min to 385 min. And the blood loss was less than 150 mL in all patients. No major complications were observed including anastomotic leakage, stenosis, or hemorrhage. Although surgical-site infection was shown in 2 cases and stasis in one case [13], all recovered with conservative treatment. 2 mm mini-loop retractor [13, 14], flexible laparoscope [13, 15], and curved instruments [14, 15] were used in most of them.

### 3.3. SILS in Other Reports with Gastric Diseases

A case about SILS perforated gastric ulcer repair was reported [18]. The operation lasted 108 minutes. The blood loss was 86 min. No additional trocars needed to be inserted and flexible laparoscope and curved reusable instruments were used in this patient.

Furthermore, the method of intracorporeal circular-stapled Billroth I anastomosis for 20 cases that underwent SILS distal gastrectomy was introduced in 1 study [19]. The average operative time was 279 (208~319) min. Neither postoperative complications, including anastomotic leakage and stricture, nor postoperative mortality was observed in this study.

Another method for intracorporeal anastomosis for gastrojejunostomy was performed in 2 cases with gastric outlet obstruction [20].

There were no cases converted to conventional (multiport) laparoscopic or open operations in all above patients.

## 4. Discussion

Since the introduction of laparoscopy in 1910 [21], the benefits of minimally invasive surgery have expanded its applicability to the treatment of benign and malignant gastric diseases [22]. Many researches have suggested that the laparoscopic approach to gastric GISTs or other benign gastric diseases is safe and effective, resulting in a short hospital stay duration and low morbidity [23, 24]. Although the radicalness and safety of laparoscopic tumor resection are controversial, there have been some retrospective studies which confirmed that laparoscopic radical gastrectomy for gastric cancer can achieve the same effect with open operation [3, 4, 25]; even laparoscopic operation is superior to the traditional operation in the matter of postoperative hospital stay, infection of incision, and postoperative complications [5, 26–28].

SILS was described by Pelosi in 1992; a single-puncture laparoscopic appendectomy was performed successfully [29]. With the popularization and development of laparoscopic technology in recent years, the application of SILS has become the current clinical research hot topic. The potential advantages of this approach are always related to the scarless healing of the incision and less wound-related complications, including bleeding, infection, and hernia.

But since the first publication about single-incision laparoscopic sleeve gastrectomy reported by Saber et al. [30], only few sporadic reports about single-incision laparoscopic gastric surgery have appeared worldwide. As the result of this review showed, only a total of 31 patients in 7 studies underwent SILS partial resection of the stomach or gastric wedge resection for gastric submucosal lesions. Simple SILS subtotal gastrectomy and perforated gastric ulcer repair were performed in 1 patient with gastric ulcer, respectively. And SILS subtotal gastrectomy with lymphadenectomy was also performed for 14 patients with early gastric cancer. Though the number of the cases was small and only the sporadic case reports were reported and reviewed, the successful SILS gastric operation itself was exciting.

SILS partial resection of the stomach or gastric wedge resection was well performed in these reports with no perioperative complications. The large volume and the quite mobile stomach made it easier. When the greater and lesser curvatures have been freed from surrounding attachments, the submucosal lesions can be quickly and accurately located especially by intraoperative endoscopy. And they also can be resected easily by Endo-GIA. Ranges of operative times were from 64 to 147 minutes and estimated blood losses were minimal. Although factors affecting operative time including the location and size of the tumor were not controlled for in Otani's study, there was no significant difference in operative time between SILS (89 min) and Otani et al. previously reported operative time for conventional laparoscopy (131 min) [31].

Also the patients were selected based on the tumor size, location, and growth morphology. For the submucosal benign diseases, the lesion's location was limited at the body of the stomach. When the lesion located at the cardia increased the difficulty of the operation, intragastric wedge resection by SILS was performed [6]. In Henckens et al.'s preliminary experience with less partial gastrectomy [9], larger or full-thickness tumors in close proximity to the pylorus often require a more formal resection (i.e., antrectomy and gastrojejunostomy) due to the high probability that a wedge resection will result in the narrowing of the distal stomach, causing iatrogenic gastric outlet obstruction. Masses in the proximity of the gastroesophageal junction can be managed similarly to tumors near the pylorus. It needs to be said that the resection of a tumor at these anatomic sites would be more difficult by laparoscopic single-site surgery. And a conventional laparoscopy or even open resection in these cases should be considered.

Adequate lymph node dissection is a complex and highly difficult operation in radical gastrectomy with gastric cancer. When performed by SILS, the difficulties appreciably increased owing to the conflict between the laparoscope and instruments inserted through the same single-incision and the lack of the operative field exposure with assistance. In early gastric cancer, lymph node involvement is infrequent and when present it predominantly involves perigastric nodes. There is, therefore, current acceptance of laparoscopic resection for early gastric cancer. Fourteen patients diagnosed as gastric early cancer were confirmed by preoperative evaluation and final pathology. Despite the longer operative time needed, there were no serious complications in these clinical reports. The number of harvested lymph nodes for malignant cases was from 12 to 67 and surgical margins were negative in all cases. Although the fewer cases and the short observation time still needed to further evaluate the benefits of this new minimally invasive technique, it is feasible and safe when performed by experienced laparoscopic surgeons.

No major short-term complications were observed including anastomotic leakage, stenosis, or hemorrhage in all these patients. Wounding bleeding was shown in one case with SILS partial gastrectomy [6] and surgical-site infection in 2 cases and stasis in one case with SILS distal gastrectomy [13]; all these patients recovered with conservative treatment. Unfortunately, no comparative studies were found in this review to compare the short-term and long-term complications with conventional laparoscopic surgery. In Lee et al.'s [32] report, the porcine model was used to compare the perioperative outcomes of SILS and conventional laparoscopic distal gastrectomy with D1+b lymph node dissection. The single-incision laparoscopic group was associated with a significantly longer operation time, but it had a similar mean number of resected lymph nodes and with similar inflammatory reaction and complication rates when compared with conventional laparoscopic. A recent meta-analysis demonstrated that single-incision laparoscopic cholecystectomy is a safe procedure for the treatment of uncomplicated gallstone disease, with postoperative outcome similar to that of standard multiport laparoscopic cholecystectomy [33]. Chakravartty et al. [34] compared 111 patients that underwent SILS-AGB (adjustable gastric banding) and 99 patients that underwent LAGB and reported that in the SILS-AGB group the median operating time (70 min) was not significantly longer than in the LAGB group (61.5 min, *P* = 0.07). However, SILS-AGB patients used less opiates (*P* < 0.01) than the LAGB patients. There was no difference in morbidity, mortality, or readmission rates.

However, three trocars at the vertex of inverted equilateral triangle about 10 mm apart may avoid collision with each other slightly. The laparoscope with optical flexible 30 degrees, flexible or curved instruments, a 2 mm mini-loop retractor, and an additional 2 mm subcostal port were helpful to facilitate surgery SILS and decrease operative time. Auxiliary traction sutures and intra-abdominal retractors can make up the shortage and expose the operative field clearly as much as possible.

In conclusion, the initial experience with single-incision laparoscopic partial gastrectomy or radical gastrectomy for gastric benign and early malignant diseases is feasible and safe when performed by experienced laparoscopic surgeons. As a result of the fewer cases and the short observation time in this group, it is still needed to further expand the number of the case and extend the follow-up time to determine the benefits of this new minimally invasive technique.

## Figures and Tables

**Table 1:** Demographic data, morphologic characterization of the lesion, and perioperative parameters.

Author	Number of patients	Diagnosis	Operation	Operative time (min)	Complication	Hospital stay (day)	Tumor size (cm)	Tumor location
Na et al. [6]	7	GIST (5) Leiomyoma (2)	Intragastric wedge resection	83.6 (70–105)	Wound bleeding	5.7 (4–7)	2.7 (2.3–3.8)	C: 4; F: 2; BL: 1

Wu et al. [7]	13	GIST	Partial resection of the stomach	99.6 (80–120)	No		0.6~5.4	F: 5; A: 1; P: 2; BL: 3 BG: 2

Hirano et al. [8]	1	GIST	Partial resection of the stomach	64	No	7	3.9	A: 1

Henckens et al. [9]	1	GIST	Gastric wedge resection	140	No	4	2	P: 1

Dapri et al. [10]	1	Stromal tumor	Gastric benign tumor resection	150	No	5	4.0 × 2.0 cm^2^	C: 1

Sasaki et al. [11]	3	GIST (2) Benign carcinoid tumor (1)	Gastric wedge resection	86 (40–147)	No	5 (4–6)	3.4 (2.4–4.0)	A: 2 BG: 1

Takahashi et al. [12]	5	GIST (4) Nonspecific inflammatory (1)	Partial resection of the stomach	89 (72–129)	No	10 (9–12)	3.6 (2.5–4.5)	F: 4 B: 1

F: fundus of stomach; C: cardia; B: body of stomach; L: lesser gastric curvature; G: greater gastric curvature; A: the anterior wall of stomach; and P: the posterior wall of stomach.

**Table 2:** Required materials of single-incision laparoscopic partial gastrectomy.

Author	Patient's position	Port system			Laparoscope			Instrument	
		Single port	Trocars	Additional port	Tip	Diameter	Degree	Graspers	Retractor
Na et al. [6]	Supine position	4-channel OCTO port V2-B		2 mm (1)	Not mentioned	10	30	Curved	Extra small Alexis wound retractor
Wu et al. [7]	Supine posture with legs apart	No	3 (10, 5, 12)	No	Rigid	10	30	Rigid	No
Hirano et al. [8]	Anti-Trendelenburg position	No	3 (5, 5, 12)	2 mm (1)	Flexile	10	Not mentioned	Not mentioned	Mini-loop retractor
Henckens et al. [9]	French position	Triport	3 (5, 5, 12)	No	Goose neck	5	30	Double-bended	Nathanson liver retractor
Dapri et al. [10]	Not mentioned	No	∗ (11, ∗, ∗)	No	Not mentioned	10	30	Curved reusable	No
Sasaki et al. [11]	Not mentioned	No	3 (5, 5, 12)	No	Flexible	5	Not mentioned	Reticulated	Mini-loop retractor
Takahashi et al. [12]	Lithotomy position	SILS	3 (5, 5, 5/12)	No	Flexible	5	Not mentioned	Sponge spacer	Mini-loop retractor

**Table 3:** Demographic data, morphologic characterization of the lesion, and perioperative parameters.

Author	Number of patients	Diagnosis	Operation	Reconstruction	Operative time (min)	Blood loss (mL)	Pathology	Lymph nodes (*n*)	Complication	Time to flatus (day)	Start regular diet (day)	Hospital stay
Omori et al. [13]	7	Early gastric cancer 1: colon CA 2: GB stone	DG + D1*β* 1: colectomy 2: LC	Roux-en-Y: 1 Billroth I: 6	344 (282–385)	50 (0–100)	cT1N0	67 (total)	Surgery-site infection: 2 Stasis: 1	3	3	Not mentioned
Park et al. [14]	2	Early gastric cancer	DG + D1*β*	Side-to-side anastomosis of stomach with duodenum	270 280	80 90	1: pT1bN0 1: pT2N2	43 21/5 (+)	No	Not mentioned	water: 2, 3 semibland diet: 3, 6	6 9
Ozdemir et al. [15]	1	Early gastric cancer	DG + D1*α*	Polya-type gastrojejunostomy	140		pT1bL1V0 N0MXG3	41	No	3	5	6
Kong et al. [16]	4	Early gastric cancer	DG + D2	Side-to-side gastroenterostomy anastomosis	280 (250–310)	162 (100–150)	1: pT1aN0, 2: pT1bN0 1: pT1bN1	16 (12–21)	No	Not mentioned	Not mentioned	13
Nonaka et al. [17]	1	Intractable gastric ulcer	DG	Roux-en-Y	412	90	H1 stage ulcer	/	No	Not mentioned	3	14

DG: distal gastrectomy.

**Table 4:** Required materials of single-incision laparoscopic distal gastrectomy and lymph node dissection.

Author	Patient's position	Port system			Laparoscope			Instrument	
		Single port	Trocars	Additional port	Tip	Diameter	Degree	Graspers	Retractor
Omori et al. [13]	Supine position with legs spread far apart	No	3 (10, 5, 12)	2 mm left subcostal midclavicular	Flexible	10	30	Rigid	2 mm mini-loop retractor;
Park et al. [14]	Not mentioned	Three-lumen single-port trocar OCTO	3 (5, 12, 12) 4 (5, 5, 5, 12)	2 mm left subcostal area	Rigid	5	30	Prototype curved instruments	2 mm needle trocar
Ozdemir et al. [15]	Reverse Trendelenburg with legs in the Lloyed Davies position	Olympus Quadport	Not mentioned	No	Flexible tip; straight	10	30	Maryland forceps	Not mentioned
Kong et al. [16]	Supine posture with legs apart	No	3 (10, 5, 12)	No	Rigid	10	30	Rigid	No
Nonaka et al. [17]	Supine, split-leg position	No	3 (5, 5, 12)	2 mm right subcostal space	Flexible	5	Not mentioned	Not mentioned	2 mm mini-loop Mini-loop retractor
